# Genetics, genomics and clinical features of adenomatous polyposis

**DOI:** 10.1007/s10689-025-00460-0

**Published:** 2025-04-16

**Authors:** Jihoon E. Joo, Julen Viana-Errasti, Daniel D. Buchanan, Laura Valle

**Affiliations:** 1 Colorectal Oncogenomics Group, Department of Clinical Pathology, The University of Melbourne, Parkville, VIC Australia; 2 Collaborative Centre for Genomic Cancer Medicine, Victorian Comprehensive Cancer Centre, Parkville, VIC Australia; 3 Hereditary Cancer Program, Catalan Institute of Oncology, IDIBELL, Hospitalet de Llobregat, Av. Gran Via 199- 203, Hospitalet de Llobregat, 08908 Spain; 4 Program in Molecular Mechanisms and Experimental Therapy in Oncology (Oncobell), IDIBELL, Hospitalet de Llobregat, Barcelona, Spain; 5 Centro de Investigación Biomédica en Red de Cáncer (CIBERONC), Madrid, Spain; 6 Doctoral Program in Biomedicine, University of Barcelona, Hospitalet de Llobregat, Barcelona, Spain; 7 Genomic Medicine and Family Cancer Clinic, Royal Melbourne Hospital, Parkville, VIC Australia

**Keywords:** Hereditary colorectal cancer, Gastrointestinal polyposis, Familial adenomatous polyposis, APC, MUTYH, NTHL1, MBD4, POLE, POLD1, MMR

## Abstract

Adenomatous polyposis syndromes are hereditary conditions characterised by the development of multiple adenomas in the gastrointestinal tract, particularly in the colon and rectum, significantly increasing the risk of colorectal cancer and, in some cases, extra-colonic malignancies. These syndromes are caused by germline pathogenic variants (PVs) in genes involved in Wnt signalling and DNA repair. The main autosomal dominant adenomatous polyposis syndromes include familial adenomatous polyposis (FAP) and polymerase proofreading-associated polyposis (PPAP), caused by germline PVs in *APC* and the *POLE* and *POLD1* genes, respectively. Autosomal recessive syndromes include those caused by biallelic PVs in the DNA mismatch repair genes *MLH1*, *MSH2*, *MSH6*, *PMS2*, *MSH3* and probably *MLH3*, and in the base excision repair genes *MUTYH*, *NTHL1* and *MBD4*. This review provides an in-depth discussion of the genetic and molecular mechanisms underlying hereditary adenomatous polyposis syndromes, their clinical presentations, tumour mutational signatures, and emerging approaches for the treatment of the associated cancers. Considerations for genetic testing are described, including post-zygotic mosaicism, non-coding PVs, the interpretation of variants of unknown significance and cancer risks associated with monoallelic variants in the recessive genes. Despite advances in genetic testing and the recent identification of new adenomatous polyposis genes, many cases of multiple adenomas remain genetically unexplained. Non-genetic factors, including environmental risk factors, prior oncologic treatments, and bacterial genotoxins colonising the intestine - particularly colibactin-producing *Escherichia coli* - have emerged as alternative pathogenic mechanisms.

## Adenomatous polyposis syndromes: causal genes and clinical characteristics

Adenomatous polyposis syndromes are characterised by the development of multiple adenomatous polyps in the colon and rectum, significantly increasing the risk of colorectal cancer (CRC), including the potential to develop multiple CRCs either as synchronous or metachronous events. Depending on the underlying aetiology, patients may have an increased risk of developing extra-colonic cancers.

**Fig. 1:**
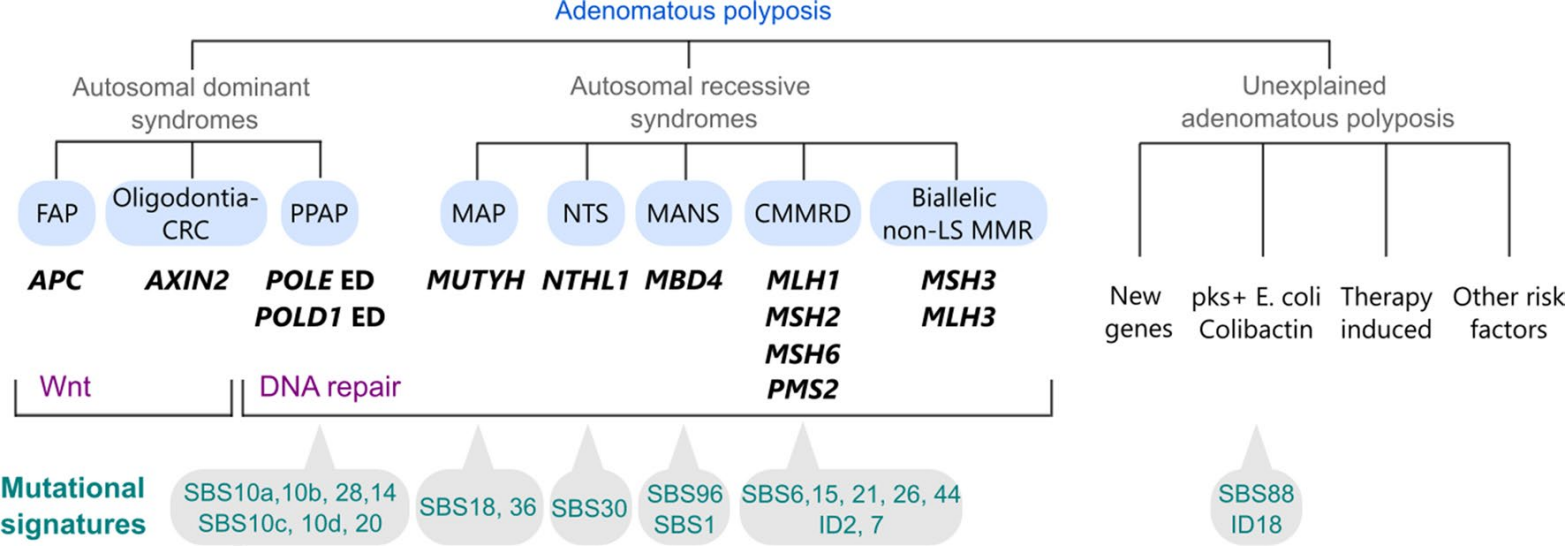
Adenomatous polyposis syndromes, mode of inheritance, causal genes, affected molecular pathways and associated COSMIC tumour mutational signatures, and possible causes of adenomatous polyposis in patients without germline PVs in known polyposis genes. *Abbreviations*: CMMRD, constitutional mismatch repair deficiency; CRC, colorectal cancer; FAP, familial adenomatous polyposis; LS, Lynch syndrome; MANS, MBD4-associated neoplasia syndrome; MAP, MUTYH-associated polyposis; MMR, mismatch repair; NTS, NTHL1 tumour syndrome; PPAP, polymerase proofreading-associated polyposis; ED, exonuclease domain

Adenomatous polyposes are distinguished from other polyposis syndromes including serrated polyposis syndrome, the hamartomatous polyposis syndromes Peutz-Jeghers, Juvenile Polyposis and PTEN-hamartoma-tumour, and mixed polyposis, by the morphology of the predominant polyp type and the underlying causes [1]. Adenomatous polyposis syndromes are primarily caused by the (dominant or recessive) inheritance of constitutional pathogenic variants (PVs) in several genes involved in Wnt signalling and DNA repair mechanisms (Fig. 1).

### Autosomal dominant syndromes

Familial adenomatous polyposis (FAP), the best characterised and most common polyposis syndrome, has a birth incidence of around one in 8500, manifests equally in both sexes, and accounts for 0.5% or less of CRC cases. FAP is caused by monoallelic constitutional PVs in *APC*, a negative regulator of Wnt signalling [2–5]. In FAP, somatic inactivation of the wild-type allele (“somatic second hit”) leads to complete inactivation of *APC*, causing constitutional activation of Wnt signalling due to the lack of degradation of the transcriptional coactivator β-catenin, which translocates to the nucleus where it binds TCF/LEF family members and activates transcription. This eventually results in the activation of oncogenes and other cancer-related genes, triggering tumourigenesis [6]. The importance of the *APC* gene in colorectal tumourigenesis is highlighted by the fact that it is one of the most commonly somatically mutated genes in CRC [7]. Up to 30% of affected individuals present ostensibly *de novo APC* PVs and thus have no family history of the disease in previous generations. When these PVs occur post-zygotically in embryonic tissues, they result in *APC* mosaicism [8, 9]. The extent of mosaicism in colonic tissues seems to determine the severity of the polyposis phenotype in these cases [9, 10].

Clinically, the classic form of FAP is characterised by the presence of hundreds to thousands of colorectal adenomas, with a disease onset in the late childhood or adolescence, with 100% risk of developing CRC if untreated. This condition is also associated with extracolonic features, some of which are highly relevant, specifically upper gastrointestinal (GI) tumours and desmoid disease, which are the main causes of FAP-related mortality [11]. In addition to CRC, but to a much lesser extent, increased cancer risk has been observed for duodenal, gastric, pancreatic, small intestine, and pancreatic cancers, hepatoblastoma, medulloblastoma, and cribriform-morular variant of papillary thyroid cancer [12]. Also, non-malignant extracolonic manifestations may occur and help with a diagnosis. These include congenital hypertrophy of the retinal pigment epithelium (CHRPE), dermoid cysts, osteomas, dental abnormalities, benign cutaneous lesions, and adrenal masses [12].

FAP is a spectrum disease, with high variability in the clinical phenotype, including attenuated forms of the disease characterised by lower polyp burden (20–100 adenomas) and later age of onset [1, 13]. A recent analysis of the data from the Danish Polyposis Registry, comprising 311 patients with classical FAP and 134 patients with attenuated FAP diagnosed in Denmark since 1974, indicated that patients with both classic and attenuated forms of FAP were at higher overall cancer risk, including CRC risk, compared with population individuals, although these risks were not different between the two groups of FAP patients. While extracolonic cancers and mortality were higher in patients with classic FAP compared with population individuals, no statistically significant increase was observed for attenuated FAP compared with population individuals [14]. The location of PVs within the *APC* gene sequence may determine the phenotypic severity of FAP, although variability between patients exists. Exon 15 of the *APC* gene contains a mutation cluster region (MCR) linked to severe phenotypes. In contrast, PVs in the 5’ and 3’ regions are associated with less severe phenotypes.

An extremely rare gastric phenotype, termed gastric adenocarcinoma and proximal polyposis syndrome (GAPPS), is caused by germline single nucleotide PVs affecting the YY1 binding site of exon 1B (promoter) of *APC* [15]. A systematic review and meta-analysis of 113 patients with a PV in the promoter 1B region showed that GAPPS is a gastric polyposis syndrome with a substantial risk of developing gastric cancer from an early age, with a remarkable variability in clinical expression within and among families. The presence of corpus fundic polyposis in addition to a family history of gastric cancer, regardless of the age at diagnosis, should be considered suggestive of GAPPS. These patients have no other manifestations resembling FAP, including intestinal, i.e., colorectal adenomatous polyposis, or extraintestinal manifestations [16].

Polymerase proofreading-associated polyposis (PPAP) is caused by constitutional PVs in *POLE* or *POLD1*. Cancer-associated PVs occur within the exonuclease domain of polymerases ε and δ, affecting their proofreading activity, and thus resulting in a particular type of DNA repair deficiency [17].

PPAP is clinically characterised by adenomatous polyposis (< 100 adenomas), and increased risk of colorectal, endometrial, ovarian, breast, brain, and upper GI cancers. The presence of café-au-lait macules (CALMs) is often observed in PPAP patients. Typically, benign and malignant neoplasms are diagnosed in the adulthood (35–60 years of age) [18]. Several PPAP cases with particularly aggressive phenotypes have been reported in the literature, with both polyposis and cancer diagnosed early in life [19–24]. These cases are characterised by early-onset diagnoses (medulloblastomas diagnosed in early childhood, and polyposis and CRC in the adolescence or young adulthood), and the presence of CALMs and non-malignant tumours (pilomatricomas, etc.), mimicking the Constitutional Mismatch Repair Deficiency (CMMRD) phenotype. Patients with such phenotypes have been associated with specific variants, including *POLE* p.E277G, p.S297F, p.V411L, p.P436R, p.M444K, p.A456P, and p.S461T, likely to induce more severe effects on the proofreading activity of the polymerase, and with the co-occurrence of constitutional PVs in *POLE* or *POLD1* exonuclease domain and in a DNA mismatch repair (MMR) gene [19–25].

Constitutional PVs in *AXIN2*, another Wnt signalling negative regulator, have been associated with oligodontia, ectodermal dysplasia (sparse hair and eyebrows) and increased risk of adenomatous polyposis [26]. Although only a few cases have been reported, the phenotypic variability is shown to be broad between heterozygotes. Some individuals exhibit one or more clinical manifestations, while those with polyps show variation in polyp number and histologic types, with most being adenomas, although serrated polyps have also been observed [27].

### Autosomal recessive syndromes

MUTYH-associated polyposis (MAP) is a recessive adenomatous polyposis syndrome caused by constitutional PVs in *MUTYH*; a gene that encodes MYH glycosylase, which is part of the DNA base excision repair (BER) system [28, 29]. After FAP, MAP is the second most common adenomatous polyposis syndrome, estimated to occur in 1:20,000 to 1:60,000 individuals due to the presence of relatively prevalent founder mutations in different populations [30].

Clinical features of MAP are mostly restricted to the GI tract. As in other polyposis syndromes, people with MAP have a broad phenotypic variability: from mild to profuse colorectal polyposis, and in some cases presenting with CRC without a polyposis phenotype. MAP patients also have increased risk of duodenal cancer, and to a lesser extent, of non-melanoma skin cancer, ovarian, endometrial or bladder cancer. Although rare, extracolonic non-malignant features including CHRPE, thyroid nodules, benign adrenal lesions, or jawbone cysts, have been reported in MAP patients [1, 12].

Biallelic PVs in *NTHL1* and *MBD4*, also BER glycosylases, cause two different ultra-rare recessive adenomatous polyposis syndromes. NTHL1 tumour syndrome (NTS) is characterised by high risk of gastrointestinal tumours, including CRC and polyposis, endometrial cancer, and breast cancer, and increased risk of other cancer types and non-neoplastic manifestations. *NTHL1* biallelic carriers have very high risk of developing multiple primary tumours [31–33]. MBD4-associated neoplasia syndrome (MANS) causes increased risk of acute myeloid leukaemia (AML) -preceded by myelodysplastic syndrome (MDS)-, adenomatous polyposis and CRC, and to a lesser extent, of uveal melanoma and schwannomas [34, 35].

Biallelic PVs in DNA mismatch repair (MMR) genes, including four MMR genes associated with Lynch syndrome (*MLH1*, *MSH2*, *MSH6* and *PMS2*) and other recessive MMR genes such as *MSH3* or *MLH3* cause adenomatous polyposis, usually as an attenuated polyposis phenotype. Constitutional mismatch repair deficiency (CMMRD) is a severe childhood cancer predisposition syndrome caused by biallelic PVs in the Lynch syndrome-related MMR genes. Most MMR variants identified in CMMRD patients are in the least penetrant Lynch syndrome genes, i.e., *PMS2* and *MSH6*. CMMRD typically presents with haematological, brain, and gastrointestinal cancers in childhood or adolescence, with a median age of onset < 10 years. CMMRD patients are highly likely to develop multiple malignancies and at increased risk of embryonal tumours, germ cell tumours, sarcomas, ganglioneuroma, melanoma, urinary tract, prostate, breast, endometrial and ovarian cancers. CMMRD is also associated with distinctive non-neoplastic features, most commonly CALMs, other skin pigmentation alterations, and multiple developmental venous anomalies [36, 37]. Mild CMMRD phenotypes resembling Lynch syndrome in tumour spectrum and age at cancer diagnosis have been reported in patients carrying biallelic hypomorphic MMR gene variants [38].

Constitutional biallelic PVs in *MSH3* have been reported in a few adult-onset adenomatous polyposis cases [39–43]. While available information points towards mostly gastrointestinal tract phenotypes, with CRC and adenomas as the main clinical features, data is still scarce to determine if there is an increased risk of tumours in other organs. Preliminary data suggests that a similar phenotype may be linked to biallelic PVs in *MLH3* although only a small number of cases have been reported to date [44, 45].

## Considerations for genetic testing

### Somatic or post-zygotic mosaicism

As mentioned before, up to 30% of FAP patients carry *de novo* PVs in *APC*, and a relevant part of these (estimated between 10% and 25%) may be the result of post-zygotic mosaicism in *APC* [8]. As a general rule, these patients lack a family history of the disease. However, in exceptional cases, somatic mosaicism may be observed in two affected members (siblings) of the same family [46]. Mosaicism occurs when the variant arises *de novo* in embryonic tissue. The presence of the disease-causing variant in different organs or tissue types depends on the timing of the mutation during the embryonic stages. The detection of mosaicism may require lowering the variant allele frequency threshold (≤ 5%) when analysing germline sequencing data; i.e., when multi-gene panel, exome or genome sequencing data obtained from blood-derived DNA is analysed. If no variants are uncovered or are difficult to discern from low level artefacts, testing other tissues, such as normal colorectal mucosa or multiple gastrointestinal polyps is highly recommended since the mosaic mutation might not be present in haematopoietic cells, and sometimes even be restricted to the colorectal epithelium [47, 48]. If the only available non-haematopoietic tissue source consists of gastrointestinal neoplasms (adenomas or cancer), at least two independent lesions should be analysed to confirm the presence of a common *APC* variant in all of them. As most adenomatous polyps acquire somatic *APC* mutations, testing multiple neoplasms is important to differentiate somatic *APC* mutations from a likely mosaic *APC* variant. Furthermore, testing for the *APC* mosaic variant using sensitive loci-specific detection techniques (e.g. digital PCR) in different sources/lineages of DNA will further enable confirmation and differentiation of localised versus soma-wide *APC* mosaicism, which has important implications for subsequent cancer risk and risk-appropriate clinical management.

Although likely to be less common than observed with *APC*, somatic mosaicism in other adenomatous polyposis genes is also expected. Somatic mosaicism has been reported in MMR genes (Lynch syndrome) [49], and other non-adenomatous polyposis genes such as *PTEN* or *STK11* [8, 50].

### Pathogenic variants in non-coding regions and structural/copy number variants affecting APC

Another potential source of missed diagnoses in adenomatous polyposis individuals is the presence of deep-intronic variants that have aberrant effects on splicing or variants that affect regulatory regions of the gene, particularly in *APC*. Although rare, intronic PVs in other (non-adenomatous) polyposis syndrome genes have also been reported [51–53].

Several recent studies have shown that deep-intronic variants in *APC* that alter splicing may be a common cause of missed diagnoses of FAP [54–57]. The use of whole-genome sequencing or gene panels that capture the intronic regions of the genes, followed by the evaluation of potential effects on splicing using in silico predictors, helps identify deep intronic variants with potential spliceogenic effects. Subsequent RNA studies are needed to clarify the actual deleterious effect, and if so, assess a potential hypomorphic nature. DNA-RNA paired testing is a straightforward approach to identify deep intronic PVs, not only in *APC* but in all risk genes included in the panels [54].

Complex and large structural variants involving *APC*, which can originate from both homologous and non-homologous recombination events mediated by Alu elements, have also been identified in FAP patients [58–63]. Multi-modal approaches, including long-read genome and RNA sequencing, optimal mapping, and chromosomal microarray, may be required when FAP is genetically unexplained, as exemplified by a case of constitutional chromothripsis involving the *APC* locus [62], or a complex rearrangement between *APC* and *TP63* [60], both of which were undetected by standard short-read multi-gene panel sequencing.

Current clinical tests primarily involve multi-gene panel testing that utilises short-read sequencing technology due to its high accuracy, high throughput capability, and cost-effectiveness. However, these approaches have limited sensitivity for detecting structural variants, particularly in low-complexity regions (e.g., repetitive sequences) [64]. As a result, a significant proportion of FAP diagnoses linked to copy number alterations and complex rearrangements may be missed by current clinical testing. A small study reported that large duplications or deletions in the *APC* gene were identified in approximately 24% of patients undergoing multi-ligation probe amplification (MLPA) testing [65], although lower detection rates have been observed in other studies [66–68]. Clinical testing using whole-genome sequencing and long-read genome and/or RNA sequencing technologies (e.g., Oxford Nanopore Technologies [69], single-molecule real-time sequencing [70]) could improve the detection of this type of variants.

The effect of certain non-coding variants on regulatory regions of the gene has been less studied, probably due to the difficulty in demonstrating their actual effect on the allelic expression. The easiest to identify are the ones located in promoter regions. Variants in the promoter 1B of *APC* have been identified in individuals with FAP, and more specifically with a rare gastric phenotype (GAPPS) [15, 71]. The recent identification and characterisation of a likely pathogenic variant in the 5’ UTR of the *APC* gene promoter region in a multi-generational family with FAP supports the inclusion of this region in multi-gene panel testing for adenomatous polyposis cases [72]. Further investigation is warranted to assess the relevance of variants in regulatory regions of polyposis genes in adenomatous polyposis patients without a known genetic cause.

In conclusion, the expansion of current clinical testing methodologies and analytical approaches beyond short-read sequencing will enable the detection of splicing-altering and non-coding regulatory variants as well as large structural and/or complex rearrangements, thereby increasing the yield of *APC*-related diagnoses.

### Specific considerations for *POLE* and *POLD1* genetic testing

Unlike any other hereditary cancer genes, loss-of-function variants in *POLE* and *POLD1* are not associated with cancer predisposition or PPAP. Only variants that affect the exonuclease activity of the polymerases, not affecting their DNA replicative ability, are the ones that should be clinically actioned. So far, these correspond to specific missense variants within the exonuclease domain [18]. Theoretically, in-frame insertions-deletions (indels) or variants that cause an in-frame splicing defect within the exonuclease domain might also be considered potentially pathogenic for PPAP.

Constitutional loss-of-function variants and variants located outside the exonuclease domain may predispose to very rare and severe autosomal recessive or dominant congenital disorders [73–78]. When identified in cancer patients, in absence of severe congenital problems, these variants should not be taken into consideration for genetic counselling or clinical purposes.

### Variant classification

An additional challenge for the accurate identification of hereditary adenomatous polyposis syndromes is the classification of germline variants of uncertain significance (VUS). The increasing application of germline multi-gene panel testing together with an increasing number of genes assessed by these panels and less phenotype-driven decision making on who gets testing has contributed to an increasing number of VUS. Uncertainty regarding the pathogenicity of a VUS impacts clinical management and the decision to test relatives for the variant.

The InSiGHT/ClinGen Hereditary Colorectal Cancer and Polyposis Variant Curation Expert Panel (https://clinicalgenome.org/affiliation/50099/; accessed 31/1/2025) was established by the International Society for Gastrointestinal Hereditary Tumours (InSiGHT) and the Clinical Genome Resource (ClinGen) with the task of developing gene-specific recommendations for variant interpretation. The panel has recently published gene-specific recommendations, based on the guidelines of the American College of Medical Genetics and Genomics and the Association for Molecular Pathology (ACMG/AMP) [79], for the classification of variants in *APC* [80], representing gene- and disease-informed specifications. The criteria were applied to 10,228 unique *APC* variants with 41% and 61% of the VUS from ClinVar and LOVD databases, respectively, reclassified into clinically actionable classes of (likely) benign and (likely) pathogenic [81]. Current gene- and disease-informed specifications are under development for the *MUTYH*, *POLE* and *POLD1* genes, and other adenomatous and hamartomatous polyposis genes will follow to optimize clinical management and opportunities for cancer prevention. Independently from the ClinGen panel, recommendations for the classification of *POLE* and *POLD1* variants in the context of cancer predisposition have also been published [18].

### Identification of monoallelic pathogenic variants in recessive adenomatous polyposis genes

Detecting heterozygous PVs in the *MUTYH*, *NTHL1*, *MSH3*, and *MBD4* genes poses a clinical challenge for counseling monoallelic carriers about their cancer risk and for establishing rational surveillance protocols.

The identification of heterozygous PVs in *MUTYH* through multi-gene panel testing is extremely common due to the high prevalence of heterozygotes in the general population (1–2%) [82]. Previous studies have reported a small but significantly increased risk of CRC in monoallelic *MUTYH* PV carriers [83–85]. However, a recent association study involving 58,998 CRC patients and 71,171 controls showed no association with CRC risk for the two most common PVs in *MUTYH* in European populations: p.Gly396Asp (rs36053993) and p.Tyr179Cys (rs34612342) [86]. Even if a low/moderate increased risk of CRC is considered, this would occur at population screening ages [87], and therefore, should not be over-interpreted. A large pan-cancer study of 10,389 patients involving 33 different types of cancer and 117,000 healthy controls revealed associated extracolonic risks for adrenocortical carcinoma, oesophageal carcinoma, sarcoma, prostate adenocarcinoma and kidney renal clear cell carcinoma, but not for colon or rectal cancers. The extra-colonic tumours from monoallelic carriers showed somatic loss of the wildtype *MUTYH* allele and presence of the characteristic *MUTYH*-associated mutational signatures [88].

The mentioned association study in 58,998 CRC patients and 71,171 controls found no association with CRC for the most commonly reported PVs in *NTHL1*-associated polyposis: c.268C>T, p.Gln90Ter (rs150766139) and c.859C>T, p.Gln287Ter (rs146347092) [86]. Likewise, analysis of 5,942 individuals with unexplained polyposis or familial CRC found no enrichment of monoallelic *NTHL1* PVs when compared to the general population [89]. No evidence of a somatic second hit affecting the wildtype allele of *NTHL1* or evidence of the SBS30 mutational signature associated with *NTHL1* deficiency, was observed in 11 CRCs and two adenomas from monoallelic *NTHL1* carriers [89]. Several studies have suggested an increased risk of breast cancer in *NTHL1* heterozygotes [90, 91]. In a pan-cancer analysis of 11,081 patients, 39 were heterozygous for *NTHL1* PVs, which was similar to the frequency observed in gnomAD non-Finnish European controls. Moreover, only two cancers had the SBS30 mutational signature: a serous ovarian carcinoma with LOH of the *NTHL1* wildtype allele, and a prostate cancer without evidence of LOH [92]. In this study, no *NTHL1* heterozygous carriers that underwent colonoscopy were identified with polyposis (≥ 10 polyps) [92].

Heterozygous germline *MBD4* PVs are associated with genetic predisposition to uveal melanoma. In these cases, the associated tumours show loss of the wildtype *MBD4* allele and, like in neoplasms from individuals with MANS, showed elevated tumour mutation burden enriched in CpG > TpG mutations (signature SBS1/SBS96) [35, 93–96]. The limited data suggests that constitutional monoallelic inactivation of *MBD4* does not increase the risk of CRC and/or polyposis [34, 35]. However, there has been a report of a monoallelic carrier of an *MBD4* pathogenic variant that developed ~ 30 adenomatous polyps and a CRC at 42 years of age with loss of MDB4 protein expression, LOH of the wildtype *MBD4* allele and an enrichment of C>T transitions within CpG sites within the CRC tissue, consistent with biallelic inactivation of *MBD4* via a somatic second hit [97].

Available evidence for *MSH3*, although limited, suggests no increased risk of cancer in *MSH3* heterozygotes. In the mentioned pan-cancer study that included 11,081 patients, 12 carried monoallelic PVs in *MSH3*, a similar frequency observed in gnomAD non-Finnish European controls. A breast cancer and a prostate cancer developed by *MSH3* heterozygotes had *MSH3* LOH causing the loss of the wildtype allele. In those tumours that could be tested, no evidence of EMAST or loss of the MSH3 protein was detected [92], as was observed in a patient with a monoallelic deletion of multiple *MSH3* exons [41].

In summary, the cumulative evidence, including a comprehensive genome-wide association study (GWAS) of imputed rare variants in hereditary adenomatous polyposis syndrome genes [86], suggests that monoallelic PVs in the recessively inherited genes *MUTYH*, *NTHL1*, *MSH3*, and *MBD4* do not increase the risk of CRC or polyposis. However, in monoallelic carriers, a somatic second hit can lead to biallelic inactivation, however, this occurs in a tissue- or organ-dependent manner, which may explain some of the extra-colonic cancer risks observed in heterozygotes.

## Tumour molecular features

Morphologically, the adenomatous polyps from people with constitutional PVs in *APC*, *AXIN2*, *POLE*, *POLD1*, MMR genes, *MUTYH*, *NTHL1*, *MBD4*, *MSH3* and *MLH3* are indistinguishable among the different syndromes and morphologically indistinguishable from sporadic adenomatous polyps. However, neoplasms that arise in each of these adenomatous polyposis syndromes can present with unique molecular characteristics.

### Somatic second hits

For the autosomal dominant genes, somatic second hits via somatic mutations or loss of heterozygosity inactivating the wildtype allele, have been demonstrated for *APC-* and *AXIN2-*related adenomas and CRCs [98, 99]. In the case of *POLE*, tumour and normal tissue data, as well as evidence in yeast, indicate that polymerase ε proofreading deficiency is haploinsufficient, not requiring the inactivation of the second allele to promote DNA damage (hypermutation) and adenoma/cancer initiation [17, 100]. Emerging evidence suggests that *POLD1* exonuclease domain mutations are haplosufficient, requiring a second hit (LOH of the wildtype allele) or MMR deficiency to cause hypermutability [100–102], although further research is needed.

### Gene-specific tumour mutational signatures associated with DNA repair deficiencies

Unique molecular characteristics have been described in neoplasms from defective DNA repair-related polyposis involving the *MUTYH*, *NTHL1*, *MBD4*, *MSH3*, *POLE* and *POLD1* genes and the MMR genes in CMMRD (Fig. 1). Except for CMMRD-related neoplasms, tumours (adenomas and cancer) developed in the context of these particular syndromes are predominantly MMR-proficient, although a subset of tumours that develop in patients with biallelic PVs in *MUTYH* or PVs in *POLE* or *POLD1* may show MMR deficiency as a result of biallelic somatic MMR gene mutations [103–105]. In general, defective DNA repair syndromes tend to result in higher tumour mutation burden (TMB) when compared to DNA repair-proficient neoplasms.

#### MMR deficiencies

CMMRD tumours are characterised for a high TMB due to the presence of MMR deficiency. MMR deficiency may be identified using either microsatellite instability (MSI) testing (by PCR or next generation sequencing (NGS) analysis) or immunohistochemical (IHC) staining for the corresponding MMR proteins MLH1, MSH2, MSH6 and PMS2. In general, MMR-deficient tumours show specific COSMIC mutational signatures that represent the highly specific DNA repair deficiency, including signatures SBS6, 15, 21, 26, and 44, and ID2 and ID7. In CMMRD, detection of MMR deficiency in non-neoplastic tissues may be used as diagnostic test and to help interpret uncertain results from genetic testing [106].

MSH3 is also part of the DNA MMR machinery, detecting and repairing replication errors in long microsatellite repeats (≥ 2 nucleotides). While *MLH1*, *MSH2*, *MSH6* or *PMS2* deficiencies affect predominantly mononucleotide and dinucleotide DNA repeat regions, biallelic *MSH3* deficiency affects tetranucleotide repeats, referred to as EMAST (Elevated Microsatellite Alterations at Selected Tetranucleotide repeats). EMAST is characteristic of tumours that develop in the context of MSH3-associated polyposis [39]. These tumours show a similar TMB and single base substitution mutation spectra to sporadic adenomas, but the proportion of small insertion/deletion (indels) is significantly higher [107]. The limited data indicates that *MLH3*-deficient tumours do not show MSI [44, 45], and it is still unknown if they have a unique mutational signature as observed in neoplasms associated with other DNA repair defects.

#### BER deficiencies

Despite not reaching the high mutational burdens of MMR- or polymerase proofreading-defective tumours, BER-deficient tumours show elevated mutational burdens and glycosylase-specific mutational signatures. *MUTYH*-deficient adenomas and cancers developed in the context of MAP are characterised by COSMIC mutational signatures SBS18 and SBS36, related to an abundance C>A nucleotide transversions [103, 108–111]. *NTHL1*-deficient tumours harbour the highly specific signature SBS30, associated with an enrichment of C>T nucleotide transitions [32, 103, 112]. The high discriminatory accuracy of these mutational signatures have shown to be particularly useful for reclassifying VUS in these genes and has better characterised the tumour spectrum of the associated syndromes.

MBD4 preferentially binds to 5-methylcytosine CpG:TpG mismatches which are the primary product of deamination at methyl CpG sites [113]. Biallelic loss of *MBD4* causes accumulation of G:T mismatches at CpG dinucleotides giving rise to excessively high levels of COSMIC mutational signature SBS1, or the highly similar SBS96 [35, 114], typically observed as having > 60% of single nucleotide variants being mCpG>TpG [34].

#### Polymerase proofreading deficiency

The presence of PVs in *POLE* and *POLD1* affecting the exonuclease activity of polymerases ε and δ, respectively, causes uncorrected errors during DNA replication resulting in the highest tumour mutational burden, often exceeding 100 mut/Mb, and referred to as ultra-hypermutator phenotype. The pattern of somatic mutations is dominated by C>A transversions that are recognised as the COSMIC mutational signature SBS10, which has been further differentiated as signatures associated with *POLE* deficiency (SBS10a, SBS10b and SBS28) or *POLD1* deficiency (SBS10c and SBS10d). When proofreading deficiency co-exists with MMR deficiency, the tumour mutational spectra shift to SBS14 in the case of *POLE* mutations and SBS20 for *POLD1* [17, 18].

## Precision oncology in the context of adenomatous polyposis syndromes

Immunotherapy, i.e., immune checkpoint inhibitors, has proven highly effective for the treatment of cancers with high tumour mutational burdens due to higher neoantigen loads [115]. This is particularly relevant in MMR-deficient and polymerase proofreading-deficient cancers, regardless of tumour type [116–118]. Good responses have also been observed in *MUTYH*- and *MBD4*-deficient cancers [119, 120]. Despite the lack of published evidence, similar responses may be expected for *NTHL1*-deficient cancers.

The deregulation of Wnt/β-catenin signalling is related to the initiation and progression of various types of cancers, including the majority of sporadic CRCs. Despite the pivotal role of β-catenin in cancer development and progression, the Wnt/β-catenin pathway is highly conserved and is involved in numerous physiological processes, making it difficult to selectively target this pathway without causing significant off-target effects and toxicity. Additionally, β-catenin lacks an easily druggable binding site, further complicating the development of inhibitors against this protein. Nevertheless, inhibitors, antagonists and agonists have been designed to target different components of the Wnt signalling pathway [121]. Although the focus on Wnt research has advanced significantly, no Wnt-targeted treatments have yet been approved. There are, however, several preclinical investigations and clinical trials underway with molecules targeting Wnt signalling that include anti-FZD antibodies, FZD domain-containing recombinant proteins, inhibitors of β-catenin/CBP interactions, and Porcupine inhibitors [122]. Activation of the Wnt pathway in tumours can lead to an immunosuppressive tumour microenvironment, limiting the effectiveness of immune checkpoint inhibitors [123], however, this opens a therapeutic opportunity by combining Wnt pathway inhibitors with immunotherapy to enhance anti-tumour immune responses in these patients.

Readthrough of premature stop codons in *APC* appears as a promising therapeutic strategy for tumours with *APC* truncating mutations in preclinical studies. In particular, macrolide ZKN-0013 can suppress premature termination of protein translation induced by nonsense mutations in the *APC* gene, resulting in the restoration of active APC protein. In vivo efficacy of ZKN-0013 was demonstrated in the *Apc*^min^ mouse model, where it significantly reduced the number of intestinal polyps [124].

## Adenomatous polyposis with unknown genetic cause

The presence of multiple adenomas in the absence of constitutional PVs in known polyposis or CRC genes is relatively common in older screening age populations [125], likely reflecting an environmental component. While older age and male sex are non-modifiable adenoma risk factors, obesity, smoking and unhealthy (western) diet have been identified as the most significant modifiable risk factors for adenoma development [126–128]. Previous treatment with abdominopelvic radiotherapy or chemotherapy with alkylating agents for childhood or young adulthood cancer has also been associated with increased risk of polyposis of varying polyp histology [129].

Recent data suggests that the presence of intestinal Colibactin-producing *pks+ Escherichia coli* might explain a relevant proportion of genetically unexplained adenomatous polyposes [130, 131]. *Pks+ E. coli* corresponds to specific strains of the bacteria that harbour the polyketide synthases complex, known as the *pks* island [132, 133]. *Pks+ E. coli* produces colibactin, a genotoxin that induces specific patterns of DNA damage, that correspond to the colibactin-associated COSMIC mutational signatures SBS88 and ID18 [134, 135]. The colibactin-associated mutation signatures may be detected in ~ 12% of sporadic CRCs [135] and in normal colonic mucosa from CRC patients [136]. The *APC* gene is one of the key targets of colibactin-related DNA damage [134]. Specifically, the *APC* c.835-8A>G splice variant occurs in the specific nucleotide sequence context targeted by colibactin-induced DNA damage and is significantly associated with the intra-tumoural presence of *pks + E. coli* in sporadic CRCs [135, 137]. The colibactin-associated *APC* mutations have been identified in a relevant number of adenomas and tumours from patients with unexplained adenomatous polyposis, demonstrating a bacterial aetiology. In a recent study, approximately 30% of unexplained adenomatous polyposis had at least one tumour harbouring the *APC* c.835-8A>G mutation, suggesting that *pks+ E. coli* might be a key contributor underlying the aetiology of idiopathic adenomatous polyposes [130, 131].

The importance of adverse lifestyle and environmental factors on the risk of CRC has been extensively documented [138], but for adenomatous polyposis, the role of these factors has not been studied. However, it should be considered given their potential to influence the gut microbiome. The gut microbiome in FAP patients differs from that of the healthy population. It has been observed that FAP patients have bacterial biofilms enriched with *E. coli* and *Bacteroides fragilis* [139], followed by the demonstration that co-colonising *Apc*^min/+^ mice with *pks+E. coli* and enterotoxigenic *Bacteroides fragilis* accelerated tumourigenesis [140]. Similarly, transplanting *Apc*^min/+^ mice with gut microbiota from CRC-affected patients activated the Wnt pathway and increased tumourigenic rates [140]. Other gut bacteria, including *Campylobacter jejuni* and *Fusobacterium nucleatum*, also showed similar effects [141, 142]. These studies not only highlight the importance of the gut microbiome in mitigating CRC risk among FAP patients but also warrant further studies investigating the interplay between the gut microbiome and environmental exposures in the pathogenesis of genetically unexplained adenomatous polyposis.

Recent efforts to identify new adenomatous polyposis genes have led to the identification of biallelic *MCM9* variants. MCM9, with MCM8, form a helicase hexameric complex involved in DNA replication (initiation), meiosis, repair of double-strand breaks via homologous recombination and DNA mismatch repair. Recessive inheritance of PVs has been linked to adenomatous polyposis, gastric cancer, and early-onset CRC, in addition to its prior documented association with infertility in both males and females related to hypogonadism and early development of germ cell tumours [143].

*BMPR2*, in addition to predisposing to pulmonary arterial hypertension, has been postulated as a gene involved in autosomal dominant polyposis, based on the recent identification of several polyposis patients with genetic variants in this gene [144]. Likewise, *FOCAD*, and Wnt signalling genes, including Wnt negative regulators *DKK4*, *HECW1*, *ITPR3*, and *WNT9B*, have been proposed as candidate causal genes for polyposis predisposition [145–147]. The evidence gathered to date for these genes is still insufficient to include them in clinical genetic diagnostics.

## Conclusions

Adenomatous polyposis occurs in the context of hereditary genetic syndromes caused by PVs in genes involved in the Wnt signalling pathway (*APC*, *AXIN2*), or DNA repair mechanisms (*POLE*, *POLD1*, *MUTYH*, *NTHL1*, *MBD4* and several MMR genes). Germline PVs in *APC* underlie the majority of adenomatous polyposis cases, followed by biallelic *MUTYH* PVs, and to a much lesser extent, PVs in the exonuclease domain of *POLE* or *POLD1*. Each adenomatous polyposis syndrome has its own distinct phenotypic features including variations in onset age, risks for different extracolonic cancers, clinical manifestations, and tumour molecular characteristics. In this regard, DNA repair-deficient tumours exhibit gene-specific mutational signatures, which may aid in variant interpretation and classification.

Genetic testing for adenomatous polyposis can present challenges, such as the identification of somatic mosaicism, complex structural variants, and deep-intronic PVs, particularly in the *APC* gene. Another challenge is interpreting monoallelic PVs in recessively inherited genes. Over the past few decades, rapidly advancing genomic technologies have enabled the identification of bona-fide and candidate risk genes, and improved our understanding of genetic and non-genetic aetiologies. Despite the discovery of new polyposis predisposition genes, up to one-third of adenomatous polyposis patients do not receive a definitive genetic diagnosis, most of which are presumed to have a non-genetic cause. Through better molecular/mutational characterisation of neoplasms using new sequencing technologies and a deeper understanding of the gut microbiome, we anticipate continued discovery of novel molecular mechanisms associated with the development of adenomatous polyposis, paving the way for personalised medicine in these patients.

## Data Availability

No datasets were generated or analysed during the current study.
